# Supplementary material for: Reporter CRISPR screens decipher cis-regulatory and trans-regulatory principles at the Xist locus
Source: Nat Struct Mol Biol. 2025 Oct 6;32(12):2465–75. doi: 10.1038/s41594-025-01686-3 (PMC12700789; doi:10.1038/s41594-025-01686-3)
Supplement: Supplementary file 1 — Supplementary Notes 1–3. [file 41594_2025_1686_MOESM1_ESM.pdf]

nature structural & molecular biology

Supplementary information https://doi.org/10.1038/s41594-025-01686-3

Reporter CRISPR screens decipher *cis*-regulatory and *trans*-regulatory principles at the Xist locus

In the format provided by the authors and unedited

Supplementary notes

Supplementary Note 1: CRISPR screen quality controls

To detect subtle effects and comprehensively identify Xist regulators, we conducted both the TFi and TFiMini screens at a high coverage (>300-fold) and with a substantial number of guides per target TF (12 for TFi and 8 for TFiMini). To validate the technical success of our screens, we evaluated several quality control metrics.

First, we verified successful Xist induction and differentiation using qPCR and RNA-FISH alongside the primary FlowFISH readout (Extended Data Fig. 1f, 2a-b, 3a-c). The functionality of the CRISPR systems was confirmed via a positive control targeting the Xist promoter region (Extended Data Fig. 1f, 2a-b). Second, we assessed the variability of guide abundance in the sgRNA libraries, which should be low prior to cell sorting, by calculating the distribution width of the sequenced sgRNAs (Extended Data Fig. 2c, 3d). This metric is determined by subtracting the log2-transformed counts at the 10th percentile from those at the 90th percentile for each replicate fraction. A narrow distribution width (<5) indicates equal representation of sgRNAs and is strongly associated with higher reproducibility in pooled screens, making this analysis essential for confirming the robustness of our results[1]. Notably, an increase in distribution width between the unsorted and sorted fractions reflects successful enrichment of the target phenotype. In contrast, non-targeting controls (NTCs) should not exhibit such enrichment, as they are not expected to influence the target characteristic (Extended Data Fig. 2c, 3d). We further assessed screen reproducibility by measuring Pearson correlations between replicate fractions, with most exceeding $r > 0.8$, indicating high-quality results (Extended Data Fig. 2d, 3e)[2]. Additionally, we investigated the consistency across replicates and of the sgRNAs targeting the same genes (Extended Data Fig. 2f-g).

Given that the screen procedure spans 10-16 days and involves multiple medium changes, we sequenced an additional control fraction following the initial selection (Selected fraction) to monitor potential effects of extended culturing (Extended Data Fig. 1e,i)[3]. In the TFi screen, we observed notable enrichment or depletion of multiple targets prior to ABA induction, indicating leakiness of the split dCas9-KRAB system (Extended Data Fig. 2e). To exclude confounding effects, we thus employed our fully inducible CasTuner system for the validation TFiMini screen, which did not exhibit the same leakiness (Extended Data Fig. 3f-g)[4]. Importantly, the results from both screens were highly correlated, suggesting that the

leakiness of the split dCas9-KRAB system did not significantly impact our overall findings (Extended Data Fig. 3h-i). In total, 12 out of 26 activators and 7 out of 24 repressors were detected in both screens (*MAGeCK mle*, Wald.p≤0.05, Extended Data Fig. 3i).

As quality control in the reporter screens we similarly assayed the distribution width of the sequenced sgRNAs across samples and replicates. The resulting values were low (~2) and increased slightly in the sorted populations (Extended Data Fig. 7c-d). Replicate fractions exhibited generally high correlation ($r > 0.7$). Notably, the GFP^{Low} populations separated much better from the Unsorted cells than the GFP^{HIgh} populations (Extended Data Fig. 7e). We further evaluated enrichment of positive controls targeting either the minimal promoter (sgFIREWACH) or the individual screen REs (Extended Data Fig. 8a). As expected all reporters were repressed by the sgFIREWACH control, while the RE-targeting guides specifically repressed their corresponding reporters. We observed two exceptions: First, sgRE127 repressed RE57M, likely due to downregulation of endogenous *Rnf12* expression. Second, sgRE61 repressed the noRE reporter, potentially via repression of the endogenous *Jpx* locus (Extended Data Fig. 8a). Interestingly, Jpx has been described to modulate CTCF activity[5] and our screens identified CTCF as a regulator of the noRE reporter (Extended Data Fig. 8b).

Supplementary Note 2: CRISPR screen results

To validate the functionality of the initial TFi screen, we included a set of 41 Xist regulators into our library (Supplementary Table 1). This control set contained 17 factors that had been shown to regulate Xist during the differentiation of mESCs via knockdown experiments (High confidence controls). In addition, the set also included Xist interaction partners and factors that had only been linked to Xist regulation during XCI maintenance or via correlative analyses. Assuringly, a majority of the high-confidence regulators (11/17) was enriched in the TFi screen (Wald.p≤0.05) (Supplementary Table 1). Similarly to two previous screens we had performed with an Xist readout[3,6], we did not detect the Xist activator YY1. As the function of YY1 in Xist regulation is well-established[7], its absence as a hit might reflect a false negative due to the inability of the used CRISPR systems to perturb YY1. Instead, we detected NFRKB as a strong Xist activator, which acts together with YY1 as part of the INO80 chromatin remodelling complex[8].

In addition, we also detected the known activators RNF12, RIF1, Ftx, Xert, Jpx and the repressive pluripotency factors REX1, MYC and KLF4[3,9–13]. However, our analysis did not identify other pluripotency factors, which had been reported to repress Xist, including OCT4,

NANOG, ESRRB and PRDM14[6,9,13]. While our study supports a role for OCT4 as an Xist activator during differentiation, the absence of the other factors remains unclear. The screen also did not detect GATA TFs (GATA1 and GATA2 are part of the TFi Lib), which we had previously characterized as potent *Xist* activators during imprinted XCI[6]. However, their absence from the screen hits likely reflects their lack of expression during 2iL withdrawal in mESCs. For the TFiMini screen, we additionally included guides targeting 16 Xist-controlling REs that we had previously identified using a non-coding CRISPR screen[3]. Of these, 15 REs were successfully detected (Wald.p<0.05), confirming the effectiveness of our validation screen.

While our approach enables high-throughput screening of a large set of candidate genes, it has two key limitations. First, we cannot directly assess the efficiency of CRISPRi-mediated knockdown for individual targets. As a consequence, we can confidently identify positive regulators, but cannot distinguish true negatives from ineffective knockdowns. Second, the screen provides limited mechanistic insight into the mode of regulation. While the screen design specifically targets factors with DNA-binding activity, we cannot exclude that some of our identified hits regulate Xist at the post-transcriptional level, e.g. via the stability of the RNA. For example, the TFi screen detected ZFP36L1 as a repressor, a factor previously characterized as an RNA-binding protein[14].

Supplementary Note 3: Computational Methods

If not stated otherwise, computational analysis was performed in *Rstudio* (v4.2), primarily using the *tidyverse* packages (v1.3.0)[15,16].

RT-qPCR analysis

Gene expression during RT-qPCR analysis was quantified using the $2^{-\Delta\Delta Ct}$ method. Here, relative expression was calculated using the two housekeeping genes *Rrm2* and *Arpo*. Significance was assessed using an unpaired t-test between the indicated samples using [t.test(var.equal=TRUE)].

RNA-FISH analysis

Xist RNA signals were either counted manually (100 cells per condition) or detected and quantified with a custom python pipeline based on the *scikit-image* toolkit (v0.22) (Fig. 5d-g)[17]. In detail, microscope images were loaded as *numpy* arrays (v1.26.2) from .czi files using *czifile* (v2019.7.2) (https://github.com/AllenCellModeling/czifile)[18]. The DAPI and Xist signals were flattened using a maximal projection along the z-axis. The nuclei were segmented using an Otsu threshold on the DAPI signals[19]. Subsequently, adjacent nuclei

were separated using a watershed algorithm. Nuclei touching the image borders were removed from the analysis. In addition, nuclei were filtered depending on size and eccentricity. Subsequently, the Xist signal inside the nuclei was segmented based on the maximal projections. First, the images were treated with a gaussian blur (sigma=2). Then, Xist signals were recognized using a local Niblack algorithm with a window size of 151 pixels and a k of -3.8. Here, a threshold is set for each individual pixel, depending on the intensities of the neighboring pixels (Threshold = mean(window) - k * sd(window)). Lastly, the Xist clouds were filtered on size.

For quantification, the number of segmented signals was counted in each cell individually. Only a small minority of cells (<1%) was falsely annotated with more than two Xist signals. The intensity was quantified per Xist signal by subtracting the background intensity in each nucleus (i.e. the geometric mean of the pixels outside segmented Xist signal) from the intensity within the segmented signal. A complete list of segmented Xist signals is shown in Supplementary Table 4. Significance for the Xist signal frequency was calculated using a paired t-test with [t.test(var.equal=TRUE, paired=TRUE)]. For comparing the Xist signal intensity, the signals within each condition were downsampled to guarantee equal representation of all three replicates and replicates were merged. Significance was then assessed using a Wilcoxon rank-sum test with [wilcox.test()]. To compare relative effects of the different perturbations on signal frequency and signal intensity, the percentage of Xist-positive cells or the $\log_{10}$-geometric mean of the Xist signal intensities was subtracted from the equivalent values for the non-targeting control (Fig. 5g).

Flow cytometry analysis

Flow cytometry data was analyzed using the *opencyto* (v.1.24.0) and *flowCore* (v1.52.1) packages[20,21]. Live cells were detected using the sideward and forward area scatter. Height and width of the forward and sideward scatters were used to discriminate singlets from doublets. Mean fluorescence intensity (MFI) was calculated as the geometric mean of the intensity in the target population minus the geometric mean of the intensity in a non-fluorescent control.

CRISPR library design

To design a CRISPR library targeting expressed TFs during early differentiation of female mESCs (TFi Lib), a list of mouse TFs was obtained from the *AnimalTFdb3.0* (Extended Fig. E1a)[22]. Next, TF genes were selected depending on their maximal expression between day 0 and 4 of 2i/LIF-withdrawal in a published RNA-seq time course of the TX1072 A3 cell line (TPM≥10) (Extended Fig. E1b)[23]. Additionally, a set of previously identified Xist regulators

was added as positive controls (Supplementary table 1). Using previously generated TT-seq data, active TSSs of these genes included in the *GENCODE M25* annotation were annotated at days 0, 2 and 4 of 2i/LIF-withdrawal[3,24]. Here, *Rsubread* (v2.0.1) was used to count reads 2 kb up- and downstream of the TSSs with [featureCounts(isPairedEnd = TRUE, strandSpecific = 2, allowMultiOverlap = TRUE)][25]. TSSs exceeding a $\log_2$ fold change of 1 between the up- and downstream bins were classified as active. Nearby TSSs within 500 bp were combined into promoters (Extended Figure E1c). All promoters were then extended to 500 bp. The resulting set of 911 target promoters, including 570 TF genes and 32 non-TF controls, was used to generate the TFi library with the *GuideScan2* webtool[26]. Up to 12 guides per promoter were chosen according to their efficiency score. Three target promoters with less than three possible guides were removed from the library. 300 non-targeting guides were added as negative controls[27]. Finally, 5' (ATCTTGTGGAAAGGACGAAACACCG) and 3' overhangs (GTTTAAGAGCTATGCTGGAAACAGCATAGCAAGT) were added to the guide sequences. The resulting library, containing 11058 guides, was ordered as an oligo pool from GenScript.

A second CRISPR library was designed to conduct a validation screen targeting Xist and to perform the reporter screens (TFiMini Lib). All TF genes that were enriched in the $Xist^{High}/Xist^{Neg}$ comparison of the TFi screen were included (*MAGeCK mle*, Wald.p≤0.2). Additionally, Nanog, Prdm14, Yy1 and Ctcf were added as targets, as they had been previously implicated in Xist regulation[7,9,28,29]. For each gene, the top 8 guides by absolute fold change were included, targeting the most significantly enriched or depleted promoter (by p-value). The 100 non-targeting controls with the lowest absolute fold change in the TFi screen were added as negative controls. Furthermore, the top 10 guides targeting selected REs at the *Xist* locus from a previous CRISPR screen were added to serve as positive controls for the reporter screen[3]. Lastly, we used the *CHOPCHOP* webtool to generate 10 guides targeting the *Fgf5* minimal promoter of the FIREWACh construct[30,31]. 5' (ATCTTGTGGAAAGGACGAAACACCG) and 3' overhangs (GTTTAAGAGCTATGCTGGAAACAGCATAGCAAGTAATGGACATCTTATTCACAG) were added to the guide sequences. The resulting library, containing 1270 guides, was ordered as an oligo pool from GenScript (Supplementary Table 1).

FlowFISH CRISPR screen analysis

Data processing

Following sequencing of the TFi and TFiMini screens, the *MAGeCK* analysis toolkit (v0.5.9.3) was used to align .fastq files to the respective CRISPR libraries and generate guide count tables with [mageck count --norm-method control] (Supplementary Table 1)[32].

Enrichment between different populations was calculated by using *MAGeCK* (v0.5.9.3) with the normalized count tables and [mle --permutation-round 10].

Quality control

Variance between the guide counts in each population was quantified as a $\log_2$ distribution width[1]. A high value suggests that the target coverage was lost during the protocol. Enrichment of a population should increase the distribution width slightly in the targeting guides, but not in the non-targeting controls. To this end, the normalized counts for each of the samples were first split between non-targeting controls and targeting guides. Next, the 10th percentile of the counts was subtracted from the 90th percentile and $\log_2$ transformed. The analysis showed that a high coverage was retained in all replicates of all screens since the $\log_2$ distribution width remained low (~1.5-3) (Supplementary Table 1). In general, the pre-sorting fractions displayed lower distribution width than the sorted fractions. This was much less pronounced in the distribution width of the non-targeting controls. To verify the reproducibility of the screens, the Pearson correlation coefficient was calculated for each population between the different replicates. Here, the normalized counts of all guides were used with [cor()]. A summary of the published controls included in the screen is listed in Supplementary Table 1.

RNA-seq analysis

Alignment and data processing

Reads were aligned single-end to the mouse reference genome (mm10) using *STAR* (v2.7.9a) with options [--outSAMattributes NH HI NM MD][33]. One of the samples (XO_36h_R1) was removed due to very low read counts. Gene expression was quantified using the GENCODE M25 annotation supplemented with the *Xert* coordinates[3,24]. Here, *Rsubread* (v2.0.1) was used with the options [featureCounts(isPairedEnd=FALSE, GTF.featureType="exon", strandSpecific=1][25]. Afterwards, counts were converted to counts per million (CPM). Mapping statistics and quality control metrics for data produced in this study are listed in Supplementary Table 2.

Integration of in vitro and in vivo RNAseq

To identify the exact stages corresponding to our *in vitro* setup, the RNA-seq time course data was integrated with a published *in vivo* scRNA-seq dataset of developing mouse embryos[34] using the *scATAcat* pipeline[35]. The scRNAseq data was retrieved from GSE100597, paired-end aligned to the mm10 reference genome and reads quantified using *STAR* (v2.7.9a) with [--outSAMattributes NH HI NM MD –quantMode GeneCounts][33]. ICM

cells at E3.5, as well epiblast cells at E4.5, E5.5 and E6.5, were identified based on the expression of marker genes and summarized in pseudobulk. Subsequently, differentially expressed genes were identified across the *in vitro* bulk samples using *DESeq2* (v.1.42.0) by conditioning on genotype and time point[36]. Each time point was compared against the control (0h) and the differentially expressed genes (Wald.FDR≤0.05) were combined to determine the final gene set. These genes, serving a similar purpose to variable genes during standard scRNA-seq analysis, were used to perform PCA with the *in vitro* and *in vivo* samples. Subsequently, all samples were visualized in the same low-dimensional space using the first two principal components.

Clustering expression dynamics across TF genes

The RNA-seq time course experiment (Fig. 3) was used to identify groups of TF genes with similar expression dynamics during 2i/LIF-withdrawal (Supplementary Table 2). Expression data from both cell lines (XX and XO) across all timepoints were used for clustering. To this end, the normalized count tables were filtered for all TF genes included in the TFi screen. Next, the counts were scaled using a z-score transformation. The TF genes were sorted according to their maximal expression compared to high Xist expression into naive (0h), early (10-30h) and late factors (36-96h). Subsequently, *k-means* clustering was used with [kmeans(n = 2)] to further separate the early (into *Transient 1* and *Transient 2*) and late factors (Into *Transient 3* and *Committed*). The enrichment of Xist activators and repressors in the different groups was quantified using a one-sided Fisher's exact test with [fisher.test(alternative="greater")].

Xist activators were primarily enriched in the three transient clusters (22/26). X-dosage sensitive expression dynamics of activator groups was analyzed depending on RNA-seq cluster or TFi screen results ($Xist^{High}$ + $Xist^{Low}$ vs $Xist^{High}$ only). To this end, the z-scores were visualized separated by genotype using a local smooth with [geom_smooth(method="loess", se=TRUE)].

Expression patterns of the transient cluster were further verified using the published *in vivo* scRNA-seq data described above (GSE100597)[34]. To this end, cells were sexed depending on the presence of selected Y-chromosomal genes that are expressed during peri-implantation (*Kdm5d*, *Zfy1*, *Zfy2*, *Eif2s3y*, *Ddx3y*, *Uty*). The single cells were then pseudo-bulked by sex and by time-point and expression of the Xist activators quantified as z-scores. Significance was assessed between sexes using a *paired t-test* ($p<0.05$).

Statistical analysis

X-dosage and differentiation sensitive TF genes were determined for the entire time course using *ANOVA* with [aov(norm_counts ~ genotype*timepoint)], followed by multiple testing correction (FDR≤0.05). Differential expression between the cell lines at individual time points was quantified using *DESeq2* (Wald.FDR≤0.05)[36].

Reporter time course analysis

For each sample the MFI was calculated as described above. Each RE sample was then normalized by calculating the $\log_2$ fold change to the noRE reporter line. This was done to control for changes in basal reporter transcription across time points. The mean MFI was then calculated as the geometric mean across replicates.

Reporter screen analysis

Count tables were generated similarly to the Xist FlowFISH screens using *MAGeCK* (v0.5.9.3) with [mageck count –norm-method control][32]. 39 Guides with low expression in the unsorted population (≤150 mean normalized counts) were removed from the analysis. Guides targeting *Zfp866* were removed from the analysis since their sequence overlapped with guides targeting *Gm20422*. Only guides with more than 150 mean counts in the unsorted samples were used for analysis. Detailed results are listed in Supplementary Table 3.

Quality Control

Variance between the guide counts and reproducibility between replicates was quantified as for the FlowFISH CRISPR screens. A high coverage was retained throughout the different reporter lines and replicates (~1.8-3.3 $\log_2$ distribution width). Replicate populations generally correlated well (~0.6-0.94 Pearson correlation coefficient).

Analysis of positive controls

To confirm the technical success of the assay, the library design included positive control guides targeting the minimal promoter of the reporter construct or the sequence of the integrated REs. Enrichment was quantified as the $\log_2$ fold change between the normalized counts in the GFP^{HIgh} or GFP^{Low} populations. Guides targeting RE57 were assigned to RE57L/M/R and RE58 according to their genomic location. Only a single guide was targeted towards RE57L.

Analysis of the noRE reporter screen

The noRE reporter screen was analyzed separately in order to identify interactions of targeted TFs with the FIREWACh minimal promoter. Significant enrichment or depletion was detected using an unpaired t-test between the normalized counts in the GFP^{High} and GFP^{Low} populations, followed by multiple testing correction (FDR<0.2). 7 TF targets (Nfrkb, Sall4, Ctcf, Zfp296, Zfp236, Sp1 and E2f4) were removed from further analysis due to their effect on the noRE reporter line (Supplementary Table 3).

Clustering analysis

To identify groups of reporter lines that behaved similarly during the screens, PCA was performed on the GFP^{High}/GFP^{Low} log_2 fold changes of the targeted TF genes with [prcomp(t(lfc_df))]. All non-TF controls were excluded from the analysis. The reporter lines were split into noRE-like, distal and proximal groups using *k-means* clustering on the log_2 fold changes with [kmeans(t(lfc_df), n=3)].

Quantification of TF-RE interactions

Interaction scores for the assayed TF-RE interactions were calculated as z-scores across all GFP^{High}/GFP^{Low} log_2 fold changes, as done before for CRISPR screen data[37]. This was done to correct for differences in observed effect sizes between the reporter lines. In detail, mean GFP^{High}/GFP^{Low} log_2 fold changes were calculated for all guide/reporter combinations. Each log_2 fold change was then normalized by subtracting the mean log_2 fold change across each target from the noRE reporter results. The corrected fold changes were scaled using a z-score transformation and named “interaction scores”. Significance was calculated using a one-sample t-test with [t.test(mu=0)], followed by multiple testing correction with [p.adjust()]. The analysis revealed a total of 166 significant TF-RE interactions (FDR≤0.2).

Downstream analysis

To confirm the validity of the interactions and to detect regulatory patterns, the reporter screen data was correlated to the TFi screen and RNA-seq data. First, interaction scores of the identified TF-RE interactions were compared to the $Xist^{High}/Xist^{Neg}$ comparison from the TFi screen across all (Fig. 4e) or individual reporters (Extended Fig. E9g). Similarity to the TFi screen was further assessed by ordering TF targets by their rank in the $Xist^{High}$ or $Xist^{Low}$ comparisons from the TFi screen and plotting a cumulative interaction score. For statistical analysis, an empirical p-value was estimated by calculating the mean cumulative score across all ranks and comparing it to the mean cumulative scores, when the ranks were scrambled (n=10,000). For visualization, the 99th and 95th percentile of the scrambled distributions at any particular rank is included in the plot. In addition, *GSEA* was performed

to test for an enrichment of TF gene expression clusters (see Fig. 3) among activating interactions in the reporter screen[38]. Additionally, enrichment of TF genes sensitive to X-chromosome number was tested. To this end, the TF genes were filtered for those with at least one significant interaction in the reporter screens (FDR≤0.2) and used *fgsea* (v1.24.0) with [fgsea(minSize=5, maxSize=500, nperm=1000, scoreType="pos"][39]. In addition, z-score transformed RNA-seq expression of positively interacting TF genes (see Fig. 3) across individual reporter lines (Extended Fig. E9h) or distal, proximal and noRE-like reporter clusters (Extended Fig. E9i-j) was calculated for the first 48 hours of 2iL-withdrawal. XX-specific expression for the proximal and distal RE clusters was assessed using a *Wilcoxon rank-sum test* (p≤0.05).

Motif enrichment analysis

DNA-binding motifs of reporter screen TFs were investigated to differentiate between direct and indirect interactions. To this end, *FIMO* (v5.1.1) was used with [fimo] to identify motifs from a comprehensive set of non-redundant TF motifs. Next, the motifs were filtered for TFs with at least one functional interaction in the reporter screens. The absolute interaction scores across all REs were plotted in the different groups as a cumulative distribution depending on the presence of a TF motif. In addition, the analysis was performed after subsetting for interactions involving Xist activators (see Fig. 1). Significance was calculated using a *Wilcoxon rank-sum test* (p≤0.05).

ChIP-seq analysis

Raw .fastq files (GSE56098, E-MTAB-7208) or processed .bigwig tracks (GSE58408) were retrieved from the GEO accession viewer or from the EMBL-EBI Array Express. For .fastq data, sequencing adaptors were removed using *Trim Galore* (v0.6.4) with option [--illumina] (https://github.com/FelixKrueger/TrimGalore). The trimmed reads were then aligned using *bowtie2* (v2.3.5.1) with the options [--very-sensitive -I 10 -X 2000] for paired-end and [--very-sensitive] for single-end data[40]. The resulting files were processed using *samtools* (v1.10) with options [view -f 2 -q 20] and [sort] for paired-end or [view -F 4 -q 20] and [sort] for single-end data[41]. Blacklisted regions for *mm10* and duplicated reads were removed using *bedtools* (v2.29.2) with options [intersect -v][42,43]*Picard* (v2.18.25) with options [MarkDuplicates VALIDATION_STRINGENCY=LENIENT REMOVE_DUPLICATES=TRUE] (http://broadinstitute.github.io/picard). Peaks were called using *MACS2* (v2.2.7.1) with standard options[44]

CUT&Tag analysis

Data processing

Processing of CUT&Tag data was performed as described previously[45]. In detail, sequencing adapters were removed using *Trim Galore* (v0.6.4) with option [--paired –nextera] (https://github.com/FelixKrueger/TrimGalore). The trimmed reads were then aligned using *bowtie2* (v2.3.5.1) with the options [--local --very-sensitive-local --no-mixed --no-discordant --phred33 -I 10 -X 2000][40] and processed using *samtools* (v1.10) with options [view -f 2 -q 20] and [sort][41]. Blacklisted regions for *mm10* were removed using *bedtools* (v2.29.2) with options [intersect -v][42,43]. For the analysis of sequencing tracks with the *UCSC genome browser*[46], .bam files of individual replicates were merged using *samtools* (v1.10) with [merge][41]. Duplicated fragments were removed using *Picard* (v2.18.25) with options [MarkDuplicates VALIDATION_STRINGENCY=LENIENT REMOVE_DUPLICATES=TRUE] (http://broadinstitute.github.io/picard). Peaks were called using *MACS2* (v2.2.7.1) with standard options[44] and .bigwig tracks generated using *deepTools* (v3.5.5) with options [bamCoverage -bs 10 --normalizeUsing CPM][47]. Only peaks overlapping between all three replicates for each sample were used for further analysis. *DiffBind* (v3.14.0) was used to determine differential peaks with [dba.analyze(method = DBA_ALL_METHODS)] and [dba.report(method = DBA_ALL_METHODS, contrast = 1, th = 0.05, bUsePval = T)]. Mapping statistics and quality control metrics are listed in Supplementary Table 4.

Xist locus analysis

To identify changes at *Xist*-controlling REs, reads were quantified *RSubread* (v2.0.1) with [featureCounts(isPairedEnd=TRUE][25]. Changes compared to the sgNT control were assessed using an *unpaired t-test* ($p \leq 0.05$). RE/histone mark combinations with <5 average counts across all samples were removed from the analysis.

scRNA-seq analysis

Data processing

Sequencing reads from the 10x cDNA library were mapped to the mouse mm10 genome with *STARsolo* (v.2.7.11a) with settings [soloType=CB_UMI_Simple, soloFeatures=GeneFull_Ex50pAS][33,48]. To map reads to either the reference B6 or the alternative Cast alleles, the "Diploid Genome" options of STARsolo were used together with experimentally determined single nucleotide variants from the TX1072 cell line[49]. To remove empty droplets and assign a quality metric to the mapping of allelic reads *EmptyDrops*[50] and *WASP*[51] were used within *STARsolo* (v.2.7.11a)[48]. Outlier cells were filtered using default

settings of *scater* (v1.26.1)[52]. MULTI-seq sample barcodes were extracted from the separately sequenced MULTIseq library and assigned to cells using *deMULTIplex2* (v1.0.1)[53]. Only cells with a uniquely assigned sample barcode were kept for further analysis.

To generate allele-specific count tables of X-chromosomal reads, reads were filtered for high-quality assignments by the presence of the *vW:i:1 WASP* filter read tag. Next, X-chromosomal reads were extracted and split into separate .bam files for each haplotype. The .bam files were sorted and indexed with *samtools* (v1.19.2)[41]. Count tables were generated using *featureCounts* (v2.0.6)[54] to assign reads to genes and *UMI-tools* (v1.1.4) to quantify UMI-level counts with option [--per-gene --gene-tag=XT --assigned-status-tag=XS --per-cell --umi-tag=CB --cell-tag=CB –extract-umi-method=tag][55]. Non-allelic count tables were generated in the same manner, using the unfiltered .bam file as input. Further downstream analyses were performed with *Scanpy* (v1.9.8) and *Anndata* (v0.10.5)[56,57]. Cells with ≥3% mitochondrial reads were removed. Read counts were normalized to 10,000 reads per cell and log-transformed using the [sc.pp.normalize_total] and [sc.pp.log1p] functions. To filter XO cells, cells with normalized Xist expression ≤0.5 and an X-chromosomal ratio (B6 chrX reads/total chrX reads) ≥0.85 or ≤0.15 were removed. PCA was performed using the 500 most variable genes with the functions [sc.pp.highly_variable_genes] and [sc.tl.pca].

Allelic analysis

To investigate X-chromosome silencing, cells with no allelic Xist counts and those with bi-allelic Xist expression were filtered out (Xist allelic fraction 0.2-0.8) (Extended Data Fig. 10d,o). In the remaining cells, the Xi was determined as the allele with the higher Xist count. The allelic fraction was then calculated for the entire chrX or individual genes with $Counts_{Xi}$ / $Counts_{Xi+Xa}$. Cells with misassigned Xi were removed (chrX allelic fraction≥0.6).

Dose-response analysis

To investigate the relationship between Xist levels and gene silencing, all cells exhibiting monoallelic Xist expression were included into a dose-response analysis (3,657 cells out of 4,081 cells with allelic Xist reads). To this end, the cells were grouped by Xist levels into 10 equally sized bins. Allelic ratios, indicative of silencing, were calculated X-chromosome wide and per X-linked gene displaying ≥1 counts in ≥30 cells per expression bin. Genes classified as escapees, defined by an allelic ratio difference <0.15 between the lowest and highest Xist expression bins, were excluded from the analysis. Subsequently, the median Xist expression in each bin was normalized to the highest Xist expression bin.

Next, a four-parameter log-logistic function was fit between the allelic ratios and the normalized Xist expression using the *drc* package (v3.0.1) with [drm(fct = LL.4(fixed = c(NA, 0, 0.5, NA)), start = c(1,0.25))][58]

References

1. Imkeller, K., Ambrosi, G., Boutros, M. & Huber, W. gscreend: modelling asymmetric count ratios in CRISPR screens to decrease experiment size and improve phenotype detection. *Genome Biol.* **21**, 53 (2020).
2. Li, W. *et al.* Quality control, modeling, and visualization of CRISPR screens with MAGeCK-VISPR. *Genome Biol.* **16**, 281 (2015).
3. Gjaltema, R. A. F. *et al.* Distal and proximal cis-regulatory elements sense X chromosome dosage and developmental state at the Xist locus. *Mol. Cell* **82**, 190–208 (2022).
4. Noviello, G., Gjaltema, R. A. F. & Schulz, E. G. CasTuner is a degron and CRISPR/Cas-based toolkit for analog tuning of endogenous gene expression. *Nat. Commun.* **14**, 3225 (2023).
5. Oh, H. J. *et al.* Jpx RNA regulates CTCF anchor site selection and formation of chromosome loops. *Cell* **184**, 6157-6173.e24 (2021).
6. Ravid Lustig, L. *et al.* GATA transcription factors drive initial Xist upregulation after fertilization through direct activation of long-range enhancers. *Nat. Cell Biol.* **25**, 1704–1715 (2023).
7. Makhlouf, M. *et al.* A prominent and conserved role for YY1 in Xist transcriptional activation. *Nat. Commun.* **5**, 4878 (2014).
8. Conaway, R. C. & Conaway, J. W. The INO80 chromatin remodeling complex in transcription, replication and repair. *Trends Biochem. Sci.* **34**, 71–77 (2009).
9. Navarro, P. *et al.* Molecular coupling of Xist regulation and pluripotency. *Science* **321**, 1693–1695 (2008).
10. Tian, D., Sun, S. & Lee, J. T. The long noncoding RNA, Jpx, is a molecular switch for X chromosome inactivation. *Cell* **143**, 390–403 (2010).
11. Gontan, C. *et al.* RNF12 initiates X-chromosome inactivation by targeting REX1 for degradation. *Nature* **485**, 386–390 (2012).
12. Furlan, G. *et al.* The ftx noncoding locus controls X chromosome inactivation independently of its RNA products. *Mol. Cell* **70**, 462-472.e8 (2018).
13. Navarro, P. *et al.* Molecular coupling of Tsix regulation and pluripotency. *Nature* **468**, 457–460 (2010).
14. Lai, W. S., Carballo, E., Thorn, J. M., Kennington, E. A. & Blackshear, P. J. Interactions

of CCCH zinc finger proteins with mRNA. Binding of tristetraprolin-related zinc finger proteins to Au-rich elements and destabilization of mRNA. *J. Biol. Chem.* **275**, 17827–17837 (2000).
15. Racine, J. S. RStudio: A Platform-Independent IDE for R and Sweave. *J. Appl. Econ* **27**, 167–172 (2012).
16. Wickham, H. *et al.* Welcome to the tidyverse. *JOSS* **4**, 1686 (2019).
17. van der Walt, S. *et al.* scikit-image: image processing in Python. *PeerJ* **2**, e453 (2014).
18. Harris, C. R. *et al.* Array programming with NumPy. *Nature* **585**, 357–362 (2020).
19. Otsu, N. A Threshold Selection Method from Gray-Level Histograms. *IEEE Trans. Syst. Man Cybern.* **9**, 62–66 (1979).
20. Finak, G. *et al.* OpenCyto: an open source infrastructure for scalable, robust, reproducible, and automated, end-to-end flow cytometry data analysis. *PLoS Comput. Biol.* **10**, e1003806 (2014).
21. Hahne, F. *et al.* flowCore: a Bioconductor package for high throughput flow cytometry. *BMC Bioinformatics* **10**, 106 (2009).
22. Hu, H. *et al.* AnimalTFDB 3.0: a comprehensive resource for annotation and prediction of animal transcription factors. *Nucleic Acids Res.* **47**, D33–D38 (2019).
23. Pacini, G. *et al.* Integrated analysis of Xist upregulation and X-chromosome inactivation with single-cell and single-allele resolution. *Nat. Commun.* **12**, 3638 (2021).
24. Frankish, A. *et al.* GENCODE reference annotation for the human and mouse genomes. *Nucleic Acids Res.* **47**, D766–D773 (2019).
25. Liao, Y., Smyth, G. K. & Shi, W. The R package Rsubread is easier, faster, cheaper and better for alignment and quantification of RNA sequencing reads. *Nucleic Acids Res.* **47**, e47 (2019).
26. Schmidt, H. *et al.* Genome-wide CRISPR guide RNA design and specificity analysis with GuideScan2. *Genome Biol.* **26**, 41 (2025).
27. Doench, J. G. *et al.* Optimized sgRNA design to maximize activity and minimize off-target effects of CRISPR-Cas9. *Nat. Biotechnol.* **34**, 184–191 (2016).
28. Payer, B. *et al.* Tsix RNA and the germline factor, PRDM14, link X reactivation and stem cell reprogramming. *Mol. Cell* **52**, 805–818 (2013).
29. Sun, S. *et al.* Jpx RNA activates Xist by evicting CTCF. *Cell* **153**, 1537–1551 (2013).
30. Montague, T. G., Cruz, J. M., Gagnon, J. A., Church, G. M. & Valen, E. CHOPCHOP: a CRISPR/Cas9 and TALEN web tool for genome editing. *Nucleic Acids Res.* **42**, W401-7 (2014).
31. Murtha, M. *et al.* FIREWACh: high-throughput functional detection of transcriptional regulatory modules in mammalian cells. *Nat. Methods* **11**, 559–565 (2014).
32. Li, W. *et al.* MAGeCK enables robust identification of essential genes from

genome-scale CRISPR/Cas9 knockout screens. *Genome Biol.* **15**, 554 (2014).
33. Dobin, A. *et al.* STAR: ultrafast universal RNA-seq aligner. *Bioinformatics* **29**, 15–21 (2013).
34. Mohammed, H. *et al.* Single-Cell Landscape of Transcriptional Heterogeneity and Cell Fate Decisions during Mouse Early Gastrulation. *Cell Rep.* **20**, 1215–1228 (2017).
35. Altay, A. & Vingron, M. scATAcat: cell-type annotation for scATAC-seq data. *NAR Genom. Bioinform.* **6**, lqae135 (2024).
36. Love, M. I., Huber, W. & Anders, S. Moderated estimation of fold change and dispersion for RNA-seq data with DESeq2. *Genome Biol.* **15**, 550 (2014).
37. Xu, P. *et al.* Genome-wide interrogation of gene functions through base editor screens empowered by barcoded sgRNAs. *Nat. Biotechnol.* **39**, 1403–1413 (2021).
38. Subramanian, A. *et al.* Gene set enrichment analysis: a knowledge-based approach for interpreting genome-wide expression profiles. *Proc Natl Acad Sci USA* **102**, 15545–15550 (2005).
39. Korotkevich, G. *et al.* Fast gene set enrichment analysis. *BioRxiv* (2016) doi:10.1101/060012.
40. Langmead, B. & Salzberg, S. L. Fast gapped-read alignment with Bowtie 2. *Nat. Methods* **9**, 357–359 (2012).
41. Li, H. *et al.* The Sequence Alignment/Map format and SAMtools. *Bioinformatics* **25**, 2078–2079 (2009).
42. Quinlan, A. R. & Hall, I. M. BEDTools: a flexible suite of utilities for comparing genomic features. *Bioinformatics* **26**, 841–842 (2010).
43. Amemiya, H. M., Kundaje, A. & Boyle, A. P. The ENCODE blacklist: identification of problematic regions of the genome. *Sci. Rep.* **9**, 9354 (2019).
44. Zhang, Y. *et al.* Model-based analysis of ChIP-Seq (MACS). *Genome Biol.* **9**, R137 (2008).
45. Kaya-Okur, H. S. *et al.* CUT&Tag for efficient epigenomic profiling of small samples and single cells. *Nat. Commun.* **10**, 1930 (2019).
46. Kent, W. J. *et al.* The human genome browser at UCSC. *Genome Res.* **12**, 996–1006 (2002).
47. Ramírez, F. *et al.* deepTools2: a next generation web server for deep-sequencing data analysis. *Nucleic Acids Res.* **44**, W160-5 (2016).
48. Kaminow, B., Yunusov, D. & Dobin, A. STARsolo: accurate, fast and versatile mapping/quantification of single-cell and single-nucleus RNA-seq data. *BioRxiv* (2021) doi:10.1101/2021.05.05.442755.
49. Barros de Andrade E Sousa, L. *et al.* Kinetics of Xist-induced gene silencing can be predicted from combinations of epigenetic and genomic features. *Genome Res.* **29**,

1087–1099 (2019).
50. Lun, A. T. L. *et al.* EmptyDrops: distinguishing cells from empty droplets in droplet-based single-cell RNA sequencing data. *Genome Biol.* **20**, 63 (2019).
51. van de Geijn, B., McVicker, G., Gilad, Y. & Pritchard, J. K. WASP: allele-specific software for robust molecular quantitative trait locus discovery. *Nat. Methods* **12**, 1061–1063 (2015).
52. McCarthy, D. J., Campbell, K. R., Lun, A. T. L. & Wills, Q. F. Scater: pre-processing, quality control, normalization and visualization of single-cell RNA-seq data in R. *Bioinformatics* **33**, 1179–1186 (2017).
53. Zhu, Q., Conrad, D. N. & Gartner, Z. J. deMULTIplex2: robust sample demultiplexing for scRNA-seq. *Genome Biol.* **25**, 37 (2024).
54. Liao, Y., Smyth, G. K. & Shi, W. featureCounts: an efficient general purpose program for assigning sequence reads to genomic features. *Bioinformatics* **30**, 923–930 (2014).
55. Smith, T., Heger, A. & Sudbery, I. UMI-tools: modeling sequencing errors in Unique Molecular Identifiers to improve quantification accuracy. *Genome Res.* **27**, 491–499 (2017).
56. Wolf, F. A., Angerer, P. & Theis, F. J. SCANPY: large-scale single-cell gene expression data analysis. *Genome Biol.* **19**, 15 (2018).
57. Virshup, I., Rybakov, S., Theis, F. J., Angerer, P. & Wolf, F. A. anndata: Access and store annotated data
matrices. *JOSS* **9**, 4371 (2024).
58. Ritz, C., Baty, F., Streibig, J. C. & Gerhard, D. Dose-response analysis using R. *PLoS ONE* **10**, e0146021 (2015).
