# Supplementary material for: Reporter CRISPR screens decipher cis-regulatory and trans-regulatory principles at the Xist locus
Source: Nat Struct Mol Biol. 2025 Oct 6;32(12):2465–75. doi: 10.1038/s41594-025-01686-3 (PMC12700789; doi:10.1038/s41594-025-01686-3)
Supplement: Supplementary file 2 — Reporting Summary [file 41594_2025_1686_MOESM2_ESM.pdf]

nature portfolio

Corresponding author(s): Edda G. Schulz

Last updated by author(s): 6/16/2025

Reporting Summary

Nature Portfolio wishes to improve the reproducibility of the work that we publish. This form provides structure for consistency and transparency in reporting. For further information on Nature Portfolio policies, see our Editorial Policies and the Editorial Policy Checklist.

Statistics

For all statistical analyses, confirm that the following items are present in the figure legend, table legend, main text, or Methods section.

n/a	Confirmed	
☐	☒	The exact sample size (*n*) for each experimental group/condition, given as a discrete number and unit of measurement
☐	☒	A statement on whether measurements were taken from distinct samples or whether the same sample was measured repeatedly
☐	☒	The statistical test(s) used AND whether they are one- or two-sided<br>*Only common tests should be described solely by name; describe more complex techniques in the Methods section.*
☒	☐	A description of all covariates tested
☐	☒	A description of any assumptions or corrections, such as tests of normality and adjustment for multiple comparisons
☐	☒	A full description of the statistical parameters including central tendency (e.g. means) or other basic estimates (e.g. regression coefficient) AND variation (e.g. standard deviation) or associated estimates of uncertainty (e.g. confidence intervals)
☐	☒	For null hypothesis testing, the test statistic (e.g. *F*, *t*, *r*) with confidence intervals, effect sizes, degrees of freedom and *P* value noted<br>*Give P values as exact values whenever suitable.*
☒	☐	For Bayesian analysis, information on the choice of priors and Markov chain Monte Carlo settings
☒	☐	For hierarchical and complex designs, identification of the appropriate level for tests and full reporting of outcomes
☐	☒	Estimates of effect sizes (e.g. Cohen's *d*, Pearson's *r*), indicating how they were calculated

Our web collection on statistics for biologists contains articles on many of the points above.

Software and code

Policy information about availability of computer code

Data collection	R (v4.2), Python (v3.10.10), MAGeCK (v0.5.9.3), opencyto (v.1.24.0), tidyverse (v1.3.2), flowCore (v1.52.1), Rsubread (v2.0.1), Samtools (v1.10), STAR (v2.7.9a), TrimGalore (v0.6.4), scikit-image (v0.22), numpy (v1.26.2), czifile (v2019.7.2), DESeq2 (v.1.42.0), fgsea (v1.24.0), FIMO (v5.1.1), bowtie2 (v2.3.5.1), bedtools (v2.29.2), Picard (v2.18.25), MACS2 (v2.2.7.1), deepTools (v3.5.5), DiffBind (v3.14.0), STARsolo (v.2.7.11a), scater (v1.26.1), samTools (v1.19.2), featureCounts (v2.0.6), Scanpy (v1.9.8), Anndata (v0.10.5), drc (v3.0.1).
Data analysis	https://github.com/EddaSchulz/TFiScreen_Paper

For manuscripts utilizing custom algorithms or software that are central to the research but not yet described in published literature, software must be made available to editors and reviewers. We strongly encourage code deposition in a community repository (e.g. GitHub). See the Nature Portfolio guidelines for submitting code & software for further information.

Data

Policy information about availability of data

All manuscripts must include a data availability statement. This statement should provide the following information, where applicable:

- Accession codes, unique identifiers, or web links for publicly available datasets
- A description of any restrictions on data availability
- For clinical datasets or third party data, please ensure that the statement adheres to our policy

Sequencing data generated in this study is available on GEO under accession number GSE274507. Raw microscopy data is available at doi.org/10.5281/

zenodo.12821363, doi.org/10.5281/zenodo.12821095 and doi.org/10.5281/zenodo.15617117. Flow cytometry data (.fcs files) is available at doi.org/10.5281/zenodo.12822424. AnimalTFDB3.0, used to design the TF screen library, is available at https://guolab.wchscu.cn/AnimalTFDB/#!/. The mm10 reference genome used in this study is available at https://hgdownload.soe.ucsc.edu/goldenPath/mm10/bigZips/latest/mm10.fa.gz.

Research involving human participants, their data, or biological material

Policy information about studies with human participants or human data. See also policy information about sex, gender (identity/presentation), and sexual orientation and race, ethnicity and racism.

Reporting on sex and gender	N/A
Reporting on race, ethnicity, or other socially relevant groupings	N/A
Population characteristics	N/A
Recruitment	N/A
Ethics oversight	N/A

Note that full information on the approval of the study protocol must also be provided in the manuscript.

Field-specific reporting

Please select the one below that is the best fit for your research. If you are not sure, read the appropriate sections before making your selection.

☒ Life sciences ☐ Behavioural & social sciences ☐ Ecological, evolutionary & environmental sciences

For a reference copy of the document with all sections, see nature.com/documents/nr-reporting-summary-flat.pdf

Life sciences study design

All studies must disclose on these points even when the disclosure is negative.

Sample size	Chosen based on experience with similar assays. Where feasible three biological replicates, which is the standard in the field, were included to confer robustness. For Xist CRISPRi screens and scRNAseq assays only two replicates were performed due to technical and financial constraints.
Data exclusions	For RNA-seq time course 1 repliate sample (XO_36h_R1) was excluded due to low read number. Detailed in the supplement.
Replication	All assays were performed with 2-3 biological replicates. Correlation between replicates is provided in supplement. All attempts at replication were successful.
Randomization	Not applicable, as the study only involved cell culture assays with genetically identical cells grown under uniform conditions.
Blinding	Not applicable, as treatments to cell culture assays were applied and analyzed by the same researcher using objective, automated measurements.

Reporting for specific materials, systems and methods

We require information from authors about some types of materials, experimental systems and methods used in many studies. Here, indicate whether each material, system or method listed is relevant to your study. If you are not sure if a list item applies to your research, read the appropriate section before selecting a response.

Materials & experimental systems

n/a	Involved in the study	
☐	☒	Antibodies
☐	☒	Eukaryotic cell lines
☒	☐	Palaeontology and archaeology
☒	☐	Animals and other organisms
☒	☐	Clinical data
☒	☐	Dual use research of concern
☒	☐	Plants

Methods

n/a	Involved in the study	
☐	☒	ChIP-seq
☐	☒	Flow cytometry
☒	☐	MRI-based neuroimaging

Antibodies

Antibodies used	H3K4me3 (Active Motif, Cat# 61379, Lot# 23107153), H3K4me1 (Active Motif, Cat# 39635, Lot# 21516012), H3K27ac (Active Motif, Cat# 39685, Lot# 23188112), Rabbit anti-mouse secondary Ab (Thermo FIsher, Cat# 31194). Antibody dilutions (1:100) are provided in Supplementary Table 6.
Validation	H3K4me1 and H3K27ac Abs were validated for CUT&Tag (Active Motif, https://www.activemotif.com/documents/tds/39635.pdf, https://www.activemotif.com/documents/tds/39685.pdf), H3K4me3 Ab was validated for ChIP-seq and previously published for CUT&Tag (Active Motif,https://www.activemotif.com/documents/tds/61379.pdf, https://pubmed.ncbi.nlm.nih.gov/36864748/) Secondary Ab was validated for IP applications (Thermo Fisher, https://www.thermofisher.com/order/genome-database/dataSheetPdf?producttype=antibody&productsubtype=antibody_secondary&productId=31194&version=Local)

Eukaryotic cell lines

Policy information about cell lines and Sex and Gender in Research

Cell line source(s)	TX1072 mouse embryonic stem cells (XX and XO lines, B6/Cast, Schulz lab), Lenti-X 293T cell line (Takara, Cat# 632180)
Authentication	All monoclonal TX1072 lines were NGS karyotyped in-house. Lenti-X 293T line was authenticated by Takara before purchasing (https://www.takarabio.com/documents/Certificate%20of%20Analysis/632180/632180-120716.pdf).
Mycoplasma contamination	Confirmed negative for all TX1072 lines. Lenti-X 293T line was confirmed negative by Takara before purchasing (https://www.takarabio.com/documents/Certificate%20of%20Analysis/632180/632180-120716.pdf).
Commonly misidentified lines (See ICLAC register)	No commonly misidentified lines were used in this study.

Palaeontology and Archaeology

Specimen provenance	*Provide provenance information for specimens and describe permits that were obtained for the work (including the name of the issuing authority, the date of issue, and any identifying information). Permits should encompass collection and, where applicable, export.*
Specimen deposition	*Indicate where the specimens have been deposited to permit free access by other researchers.*
Dating methods	*If new dates are provided, describe how they were obtained (e.g. collection, storage, sample pretreatment and measurement), where they were obtained (i.e. lab name), the calibration program and the protocol for quality assurance OR state that no new dates are provided.*

☐ Tick this box to confirm that the raw and calibrated dates are available in the paper or in Supplementary Information.

Ethics oversight	*Identify the organization(s) that approved or provided guidance on the study protocol, OR state that no ethical approval or guidance was required and explain why not.*

Note that full information on the approval of the study protocol must also be provided in the manuscript.

Animals and other research organisms

Policy information about studies involving animals; ARRIVE guidelines recommended for reporting animal research, and Sex and Gender in Research

Laboratory animals	*For laboratory animals, report species, strain and age OR state that the study did not involve laboratory animals.*
Wild animals	*Provide details on animals observed in or captured in the field; report species and age where possible. Describe how animals were caught and transported and what happened to captive animals after the study (if killed, explain why and describe method; if released, say where and when) OR state that the study did not involve wild animals.*
Reporting on sex	*Indicate if findings apply to only one sex; describe whether sex was considered in study design, methods used for assigning sex. Provide data disaggregated for sex where this information has been collected in the source data as appropriate; provide overall numbers in this Reporting Summary. Please state if this information has not been collected. Report sex-based analyses where performed, justify reasons for lack of sex-based analysis.*
Field-collected samples	*For laboratory work with field-collected samples, describe all relevant parameters such as housing, maintenance, temperature, photoperiod and end-of-experiment protocol OR state that the study did not involve samples collected from the field.*
Ethics oversight	*Identify the organization(s) that approved or provided guidance on the study protocol, OR state that no ethical approval or guidance was required and explain why not.*

Note that full information on the approval of the study protocol must also be provided in the manuscript.

Clinical data

Policy information about clinical studies

All manuscripts should comply with the ICMJE guidelines for publication of clinical research and a completed CONSORT checklist must be included with all submissions.

Clinical trial registration	*Provide the trial registration number from ClinicalTrials.gov or an equivalent agency.*
Study protocol	*Note where the full trial protocol can be accessed OR if not available, explain why.*
Data collection	*Describe the settings and locales of data collection, noting the time periods of recruitment and data collection.*
Outcomes	*Describe how you pre-defined primary and secondary outcome measures and how you assessed these measures.*

Dual use research of concern

Policy information about dual use research of concern

Hazards

Could the accidental, deliberate or reckless misuse of agents or technologies generated in the work, or the application of information presented in the manuscript, pose a threat to:

No	Yes	
☒	☐	Public health
☒	☐	National security
☒	☐	Crops and/or livestock
☒	☐	Ecosystems
☒	☐	Any other significant area

Experiments of concern

Does the work involve any of these experiments of concern:

No	Yes	
☒	☐	Demonstrate how to render a vaccine ineffective
☒	☐	Confer resistance to therapeutically useful antibiotics or antiviral agents
☒	☐	Enhance the virulence of a pathogen or render a nonpathogen virulent
☒	☐	Increase transmissibility of a pathogen
☒	☐	Alter the host range of a pathogen
☒	☐	Enable evasion of diagnostic/detection modalities
☒	☐	Enable the weaponization of a biological agent or toxin
☒	☐	Any other potentially harmful combination of experiments and agents

Plants

Seed stocks	*Report on the source of all seed stocks or other plant material used. If applicable, state the seed stock centre and catalogue number. If plant specimens were collected from the field, describe the collection location, date and sampling procedures.*
Novel plant genotypes	*Describe the methods by which all novel plant genotypes were produced. This includes those generated by transgenic approaches, gene editing, chemical/radiation-based mutagenesis and hybridization. For transgenic lines, describe the transformation method, the number of independent lines analyzed and the generation upon which experiments were performed. For gene-edited lines, describe the editor used, the endogenous sequence targeted for editing, the targeting guide RNA sequence (if applicable) and how the editor was applied.*
Authentication	*Describe any authentication procedures for each seed stock used or novel genotype generated. Describe any experiments used to assess the effect of a mutation and, where applicable, how potential secondary effects (e.g. second site T-DNA insertions, mosiacism, off-target gene editing) were examined.*

ChIP-seq

Data deposition

☒ Confirm that both raw and final processed data have been deposited in a public database such as GEO.

☐ Confirm that you have deposited or provided access to graph files (e.g. BED files) for the called peaks.

Data access links<br>*May remain private before publication.*	GSE273068
Files in database submission	Bigwig, fastq
Genome browser session<br>(e.g. UCSC)	https://genome.ucsc.edu/s/tillsc23/CnT_xist_act

Methodology

Replicates	3
Sequencing depth	paired-end, 100 bp, 3.5-9.3*10^6 fragments (CUT&Tag)
Antibodies	H3K4me3 (Active Motif, Cat# 61379, Lot# 23107153), H3K4me1 (Active Motif, Cat# 39635, Lot# 21516012), H3K27ac (Active Motif, Cat# 39685, Lot# 23188112), Rabbit anti-mouse secondary Ab (Thermo FIsher, Cat# 31194) - Used for CUT&Tag
Peak calling parameters	MACS2 standard options
Data quality	Nr. of peaks, FrIP and duplicate reads available in supplement
Software	https://github.com/EddaSchulz/TFiScreen_Paper

Flow Cytometry

Plots

Confirm that:

☒ The axis labels state the marker and fluorochrome used (e.g. CD4-FITC).

☒ The axis scales are clearly visible. Include numbers along axes only for bottom left plot of group (a 'group' is an analysis of identical markers).

☒ All plots are contour plots with outliers or pseudocolor plots.

☒ A numerical value for number of cells or percentage (with statistics) is provided.

Methodology

Sample preparation	Mouse embryonic stem cells, unfixed with GFP reporter (Reporter screens) or fixed with PrimeFlow kit (Xist screens)
Instrument	FACSAria Fusion flow cytometer (BD)
Software	https://github.com/EddaSchulz/TFiScreen_Paper
Cell population abundance	Not applicable (sorted cells were harvested for CRISPR screens)
Gating strategy	Gating live cells + singlets with forward and sideward scatters, then sorting GFP/Alexa 647 high and low populations

☒ Tick this box to confirm that a figure exemplifying the gating strategy is provided in the Supplementary Information.

Magnetic resonance imaging

Experimental design

Design type	*Indicate task or resting state; event-related or block design.*
Design specifications	*Specify the number of blocks, trials or experimental units per session and/or subject, and specify the length of each trial or block (if trials are blocked) and interval between trials.*
Behavioral performance measures	*State number and/or type of variables recorded (e.g. correct button press, response time) and what statistics were used to establish that the subjects were performing the task as expected (e.g. mean, range, and/or standard deviation across*

subjects).

Acquisition

Imaging type(s) — Specify: functional, structural, diffusion, perfusion.

Field strength — Specify in Tesla

Sequence & imaging parameters — Specify the pulse sequence type (gradient echo, spin echo, etc.), imaging type (EPI, spiral, etc.), field of view, matrix size, slice thickness, orientation and TE/TR/flip angle.

Area of acquisition — State whether a whole brain scan was used OR define the area of acquisition, describing how the region was determined.

Diffusion MRI ☐ Used ☐ Not used

Preprocessing

Preprocessing software — Provide detail on software version and revision number and on specific parameters (model/functions, brain extraction, segmentation, smoothing kernel size, etc.).

Normalization — If data were normalized/standardized, describe the approach(es): specify linear or non-linear and define image types used for transformation OR indicate that data were not normalized and explain rationale for lack of normalization.

Normalization template — Describe the template used for normalization/transformation, specifying subject space or group standardized space (e.g. original Talairach, MNI305, ICBM152) OR indicate that the data were not normalized.

Noise and artifact removal — Describe your procedure(s) for artifact and structured noise removal, specifying motion parameters, tissue signals and physiological signals (heart rate, respiration).

Volume censoring — Define your software and/or method and criteria for volume censoring, and state the extent of such censoring.

Statistical modeling & inference

Model type and settings — Specify type (mass univariate, multivariate, RSA, predictive, etc.) and describe essential details of the model at the first and second levels (e.g. fixed, random or mixed effects; drift or auto-correlation).

Effect(s) tested — Define precise effect in terms of the task or stimulus conditions instead of psychological concepts and indicate whether ANOVA or factorial designs were used.

Specify type of analysis: ☐ Whole brain ☐ ROI-based ☐ Both

Statistic type for inference — Specify voxel-wise or cluster-wise and report all relevant parameters for cluster-wise methods.

(See Eklund et al. 2016)

Correction — Describe the type of correction and how it is obtained for multiple comparisons (e.g. FWE, FDR, permutation or Monte Carlo).

Models & analysis

n/a	Involved in the study
☐	☐ Functional and/or effective connectivity
☐	☐ Graph analysis
☐	☐ Multivariate modeling or predictive analysis

Functional and/or effective connectivity — Report the measures of dependence used and the model details (e.g. Pearson correlation, partial correlation, mutual information).

Graph analysis — Report the dependent variable and connectivity measure, specifying weighted graph or binarized graph, subject- or group-level, and the global and/or node summaries used (e.g. clustering coefficient, efficiency, etc.).

Multivariate modeling and predictive analysis — Specify independent variables, features extraction and dimension reduction, model, training and evaluation metrics.
